# Conserved and Unique Functions of Blimp1 in Immune Cells

**DOI:** 10.3389/fimmu.2021.805260

**Published:** 2022-01-27

**Authors:** Samantha Nadeau, Gislâine A. Martins

**Affiliations:** ^1^ F. Widjaja Foundation Inflammatory Bowel and Immunobiology Research Institute (IBIRI), Cedars-Sinai Medical Center (CSMC), Los Angeles, CA, United States; ^2^ Department of Biomedical Sciences, Research Division of Immunology, Cedars-Sinai Medical Center (CSMC), Los Angeles, CA, United States; ^3^ Department of Medicine, Gastroenterology Division, Cedars-Sinai Medical Center (CSMC), Los Angeles, CA, United States

**Keywords:** transcription factor, terminal differentiation, gene regulation, repressor, PRDM1, PRDI-BF1/Blimp1

## Abstract

B-lymphocyte-induced maturation protein-1 (Blimp1), is an evolutionarily conserved transcriptional regulator originally described as a repressor of gene transcription. Blimp1 crucially regulates embryonic development and terminal differentiation in numerous cell lineages, including immune cells. Initial investigations of Blimp1’s role in immunity established its non-redundant role in lymphocytic terminal effector differentiation and function. In B cells, Blimp1 drives plasmablast formation and antibody secretion, whereas in T cells, Blimp1 regulates functional differentiation, including cytokine gene expression. These studies established Blimp1 as an essential transcriptional regulator that promotes efficient and controlled adaptive immunity. Recent studies have also demonstrated important roles for Blimp1 in innate immune cells, specifically myeloid cells, and Blimp1 has been established as an intrinsic regulator of dendritic cell maturation and T cell priming. Emerging studies have determined both conserved and unique functions of Blimp1 in different immune cell subsets, including the unique direct activation of the *igh* gene transcription in B cells and a conserved antagonism with BCL6 in B cells, T cells, and myeloid cells. Moreover, polymorphisms associated with the gene encoding Blimp1 (*PRDM1*) have been linked to numerous chronic inflammatory conditions in humans. Blimp1 has been shown to regulate target gene expression by either competing with other transcription factors for binding to the target loci, and/or by recruiting various chromatin-modifying co-factors that promote suppressive chromatin structure, such as histone de-acetylases and methyl-transferases. Further, Blimp1 function has been shown to be essentially dose and context-dependent, which adds to Blimp1’s versatility as a regulator of gene expression. Here, we review Blimp1’s complex roles in immunity and highlight specific gaps in the understanding of the biology of this transcriptional regulator, with a major focus on aspects that could foster the description and understanding of novel pathways regulated by Blimp1 in the immune system.

## 1 Introduction

**Figure 1:**
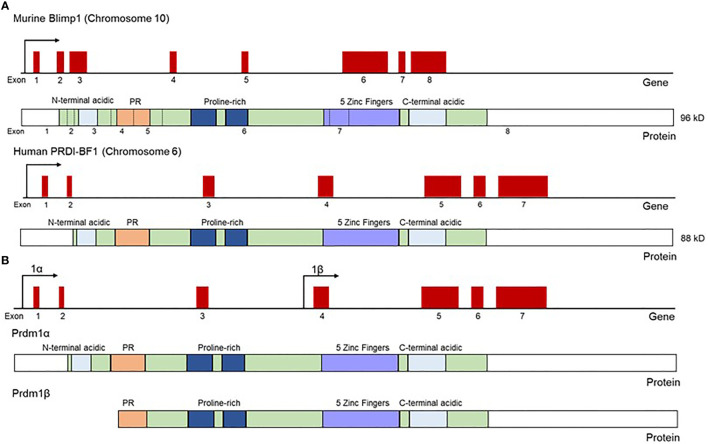
Schematic representation of Blimp1 and PRDI-BF1 mRNA and protein structure in mouse and human. **(A)** Both human and murine homologs of Blimp1 contain five Krüppel-type zinc fingers, two acidic regions (N and C terminal), proline-rich region and a PR domain. Murine Blimp1 contains 67 extra N-terminal amino acids compared to the human homolog PRDI-BF1, and Blimp1 and PRDI-BF1 are 90% identical to each other and can be used interchangeably in functional assays. Gene diagrams show exons as raised red boxes. Protein diagrams show exonic regions (green), acidic regions (light blue), PR domain (orange), proline-rich region (dark blue) and zinc fingers (purple). **(B)** The full-length Blimp1 transcript encodes Blimp1α, while a truncated transcript encoded from an alternative promoter (beta promoter) was name Blimp1β, and the resultant Blimp1β protein is 700 amino acids long and lacks the N-terminal acidic region and part of the PR domain found in the full length Blimp1α protein. Protein diagrams show exonic regions (green), acidic regions (light blue), PR domain (orange), proline-rich region (dark blue) and zinc fingers (purple). Adapted from Györy et al. (4) and Tunyaplin et al. (5).

**Figure 2:**
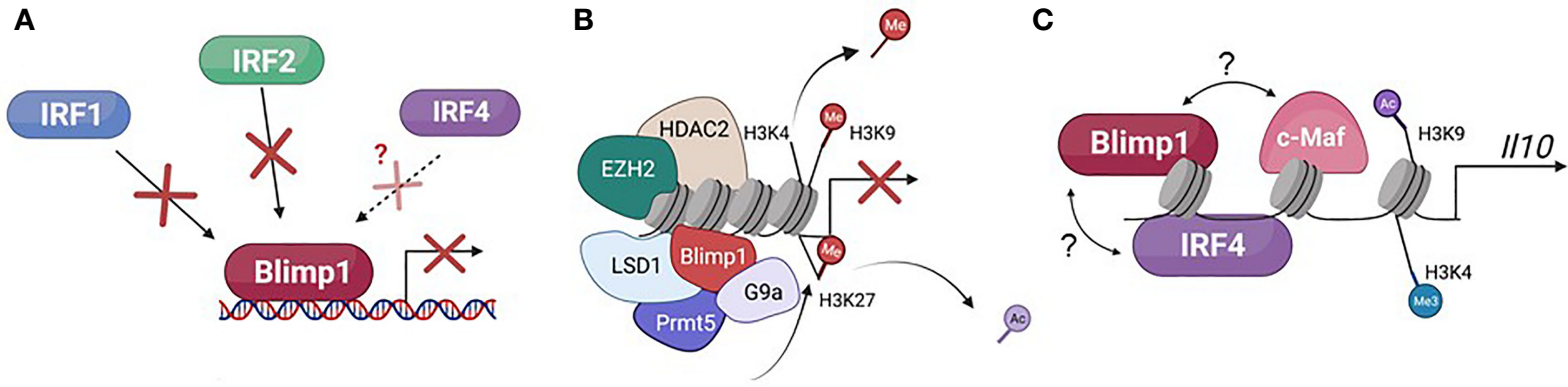
Described mechanisms of gene regulation by Blimp1. **(A)** Blimp1 can repress target gene transcription by competing with transcriptional activators, specifically IRF1 and IRF2, and potentially IRF4, for binding to different target genes. **(B)** Blimp1 can repress target gene transcription by recruiting chromatin-modifying co-factors that promote suppressive chromatin structure, notably HDAC2 (histone de-acetylase 2, de-acetylates H3), G9a (lysine histone methyltransferase, H3K9 and H3K27), EZH2 (methylates H3K27), LSD1 (histone lysine demethylase, H3K4 and H3K9), and Prmt5 (di-methylates arginine residues, H2A and H4). **(C)** Blimp1 has been shown to activate *Il10* transcription by binding directly to its locus, in a cooperative manner with IRF4, and mediating chromatin modifications that facilitate transcription (tri-methylation of H3K4 and acetylation of H3K9). Blimp1 has also been shown to cooperate with c-Maf to induce *Il10* transcription in T cells, but whether or not they physically bind with each other is not known.

The transcription factor PRDI-BF1/Blimp1, encoded by the *PRDM1* gene, was first described in human sarcoma cell lines as a repressor of the IFN-β gene (1). The observation that this *PRDM1*-encoded protein binds to the IFN-β promoter at the positive regulatory domain 1 (PRDI) region led to the acronym PRDI-BF1 (Positive Regulatory Domain 1 – Binding Factor 1). Soon after, the same protein, this time in a murine system, was named Blimp1 (B-lymphocyte-induced maturation protein 1), in work done by Mark Davis and colleagues, which revealed Blimp1’s role in driving functional differentiation and plasmablast formation in murine B lymphoma cell lines (2). This was then followed by the observation that human PRDI-BF1 and murine Blimp1 are highly similar homologs of each other (3). Both the human and the murine PRDI-BF1/Blimp1 proteins contain five Krüppel-like zinc finger DNA binding domains located at the C-terminus of the protein (**Figure 1**), the first two of which were described as necessary for binding to the IFN-β promoter (1, 2, 6). Blimp1 recruits chromatin-modifying factors, such as hGroucho and histone deacetylases, to its target locus through a proline-rich region at the N-terminal side of the zinc fingers, and through this recruitment enables repressive chromatin modifications and downregulates target gene transcription (**Figure 2**) (7, 8).

In addition to human and murine cells, Blimp1 homologs have been described in *Caenorhabditis elegans*, *Drosophila melanogaster*, *Xenopus*, sea urchin, and zebrafish. The Blimp1 homolog BLMP-1 in *C. elegans* enables proper gonadal cell migration during development (9), and the Blimp1 homolog in *D. melanogaster*, *Drosophilablimp-1*, facilitates tracheal formation during embryogenesis (10). In zebrafish, the Blimp1 homolog u-boot (ubo) enables RB sensory neuron and neural crest cell differentiation as well as the differentiation of slow-twitch muscle fibers (11, 12). Thus, Blimp1 functions in terminal differentiation not only in mammalian species but in several species that are evolutionary distant from humans and mice, and the molecular mechanisms of Blimp1 function may be conserved, to some degree, in different cell types and species.

In addition to its ever-expanding role in the immune system, discussed below, Blimp1 is also crucial for murine primordial germ cell formation, and mice with a global deletion of Blimp1 are embryonically lethal (13, 14). During pregnancy and embryo implantation, Blimp1 expression increases in the uterine epithelial cells and plays crucial roles in decidual tissue development (15). In fact, female mice with a conditional deletion of Blimp1 in progesterone-responsive tissues, mediated by crossing mice expressing CRE under control of the progesterone receptor regulatory regions (PR^CRE^) to mice with “floxed” *Prdm1* alleles (*Prdm1* ^F/F^ mice), display smaller deciduae and impaired decidual zone establishment during pregnancy (15).

In the developing small intestine, Blimp1 crucially maintains proper enterocyte maturation and development, and mice with a conditional deletion of Blimp1 in intestinal epithelial cells, generated by crossing Villin^CRE^ mice to *Prdm1* ^F/F^ mice, display impaired growth and significantly higher mortality compared to wild-type and heterozygous controls (16, 17). *Prdm1* ^F/F^-Villin^CRE+^ conditional knock-out (CKO) mice also exhibit an adult-like intestinal epithelium shortly after birth, indicating Blimp1 ensures the proper development of the intestinal epithelium from the suckling-to-weaning stage (17).

Chromatin Immunoprecipitation sequencing (ChIP-seq) analyses have revealed that Blimp1 and interferon regulatory factor 1 (Irf1) share overlapping binding sites at genes related to antigen processing and expression of MHC class I in the embryonic day 18.5 (E18.5) developing intestinal epithelium, and expression analyses suggest Blimp1 and Irf1 might compete to regulate MHCI expression and maturation of the intestinal epithelium (18). Blimp1’s non-redundant roles during embryonic development have been comprehensively reviewed elsewhere (19). In this review, we will focus on the established roles of Blimp1 as a transcriptional regulator, both as an activator and a repressor, in immune cell subsets and discuss opportunities to further the understanding of Blimp1’s biology and its role in immune homeostasis and diseases.

## 2 The Role of Blimp1 in the Immune System

### 2.1 The Requirement of Blimp1 for the Terminal Differentiation of Plasma Cells

Davis’s group discovery of Blimp1’s role in B cells was the first of an extensive body of work linking Blimp1 to terminal effector differentiation in B lymphocytes. Ectopic expression of Blimp1 in mature B cells drives plasmablast formation, indicating a crucial role for Blimp1 in B cell differentiation and antibody production (2). Work from the Calame lab showed the requirement of Blimp1 for plasma cell differentiation *in vivo* by generating mice with a conditional deletion of Blimp1 in B cells. This was achieved by flanking exons 6-8 of the *Prdm1* gene with LoxP sites (“floxed”; *Prdm1^F/F^*) and then crossing the resulting *Prdm1* ^F/F^ mice with mice bearing a CD19^CRE^ transgene, thereby preventing expression of functional Blimp1 protein in B cells (20). Using this model, it was shown that CD19^CRE^ CKO mice display depleted plasma cell populations and serum immunoglobulin (Ig) in response to both T cell-dependent and T cell-independent antigens, although these mice exhibit normal B cell development, indicating Blimp1 plays a specific and crucial role in the development of plasmablasts and antibody secretion during an immune response (20). Further work from the Calame lab showed that during plasma cell differentiation, Blimp1 directly represses the transcriptional regulators c-Myc, Pax5 and Bcl6, mitigating cell proliferation and promoting terminal differentiation (21–24). BCL6 (B Cell Lymphoma-6) promotes cell proliferation in B cells and preventing terminal effector differentiation, and BCL6 also directly represses *Prdm1* (25, 26). During plasmablast differentiation, Blimp1 downregulates cell proliferation and instead promotes endoplasmic reticulum (ER) remodeling and antibody production. In fact, Blimp1 and BCL6 act antagonistically to regulate plasma cell differentiation by functioning as transcriptional repressors of each other (5, 24).

In Blimp1-sufficient mice, plasmablasts utilize the unfolded protein response (UPR) to expand the ER, creating the machinery necessary for producing and secreting antibodies. CD19^CRE^ Blimp1CKO mice fail to repress Pax5 in B-1 B cells and, as a result, fail to upregulate XBP-1, a necessary step for the UPR and consequent ER expansion for antibody secretion, establishing Blimp1 as an integral driver of this process (22). In fact, Blimp1α, the full-length transcript of Blimp1, is induced in human B cells in an NF-κB dependent fashion upon activation of UPR pathways, whereas Blimp1β, the truncated isoform of Blimp1 that lacks the PR-domain (**Figure 1** and further discussed below), is not induced (27). The Blimp1α isoform is also induced in human myeloid cell lines as a response to UPR pathways in the same manner, indicating Blimp1 may play conserved functions in cell stress responses in both lymphoid and myeloid populations (27).

**Figure 3:**
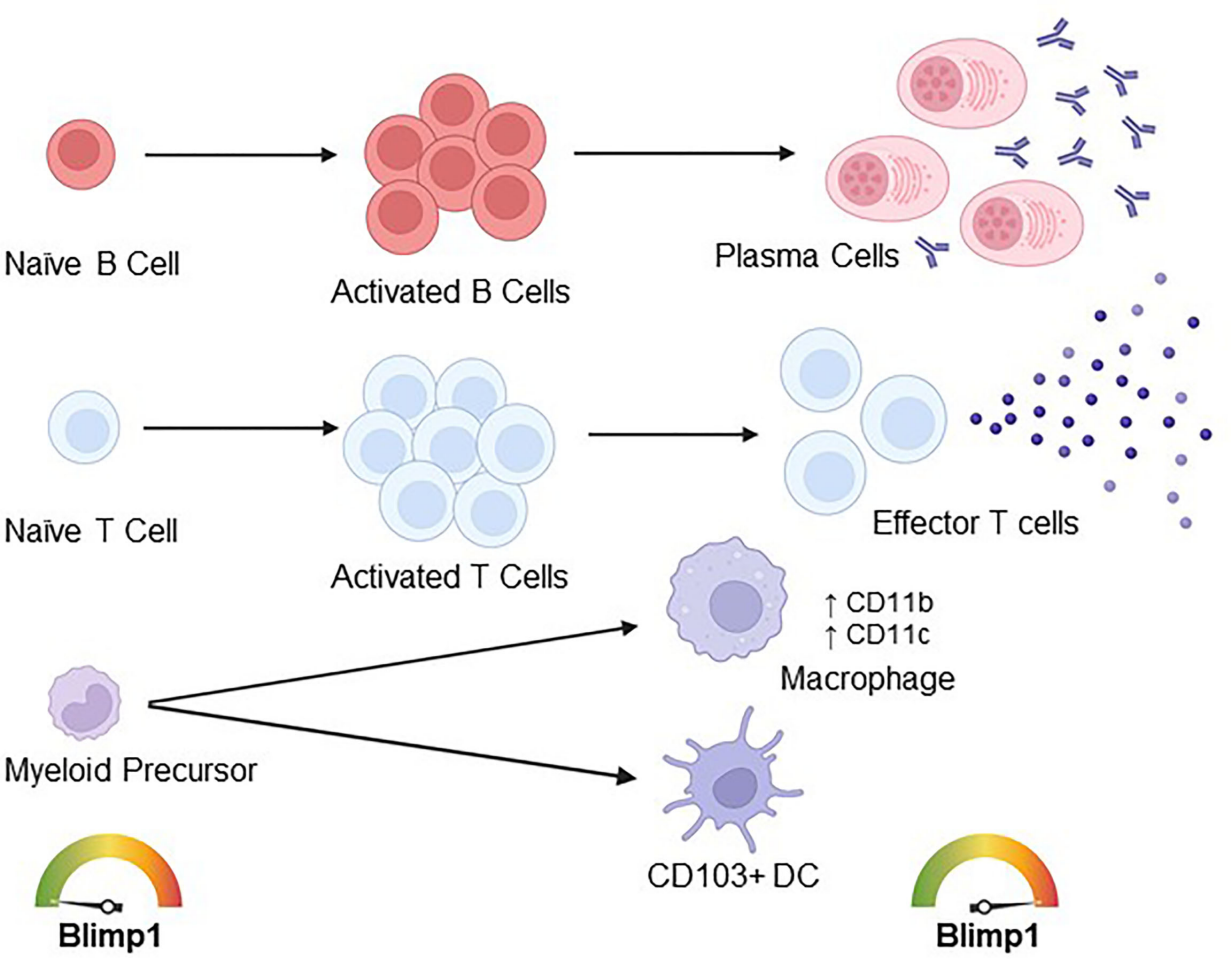
Described roles of Blimp1 in immune cell effector differentiation and function. Blimp1 expression in naïve B cells is low, however, during activation Blimp1 is upregulated and necessary for the differentiation of antibody-secreting plasma cells. Similarly, Blimp1 is lowly expressed in naïve T cells and upregulated during T cell activation, with higher expression in effector T cells. Further, Blimp1 plays important roles in effector T cell function, suppressing the differentiation of T_FH_ cells and regulating the expression of several cytokines (including *Il2*, *Il17, Il10, Ifn)*, chemokines and cytokine/chemokine receptors *(*including *Ccl8, Il-23r, Cxcr5, Il2ra, Ccr7)* and surface molecule *(*including *PD-1* and *Ctla4)* genes. In dendritic cells (DCs), Blimp1 restrains autoantibody production by mitigating IL-6 production and antigen presentation on MHCII, and Blimp1 plays a role in the differentiation of CD103^+^ intestinal DCs. Blimp1 is also upregulated during macrophage differentiation, and Blimp1 overexpression *in vitro* is sufficient to drive macrophage differentiation.

Blimp1 expression is not detected in human memory B cells, and CD19^CRE^ CKO mice retain memory B cell populations, indicating that these cells are maintained and formed in a Blimp1-independent manner. *Prdm1* ^F/F^CD19^CRE^ CKO mice also show no impairment in early B cell development in the bone marrow and production of naïve peripheral B cells. However, CD19^CRE^ CKO mice display impaired maintenance of long-lived antibody secreting bone marrow plasma cells (28). Of note, the premature expression of Blimp1 during B cell development results in accelerated plasma cell development and generation of self-reactive antibodies, resulting in the onset of autoimmune disease in aged mice, further illustrating the relevance of Blimp1 in B cell responses in general (**Figure 3**) (29).

### 2.2 Blimp1’s Role in T Lymphocytes

In addition to B cells, Blimp1 has also been extensively studied as a crucial regulator of T lymphocyte function. Similar to observations in B cells, Blimp1 is highly expressed in terminally differentiated effector T cell populations, while its expression is low in naïve T cells, indicating a conserved role for Blimp1 in mediating effector lymphocytic functional differentiation (**Figure 3**). As observed in B cells, Blimp1 also acts antagonistically to BCL6 in T lymphocytes, mitigating follicular helper T cell (T_fh_) differentiation and, thus, indirectly modulating antibody production (30). A similar antagonism between Blimp1 and BCL6 could be operative in CD8^+^ T cells, as BCL6 enables central memory CD8^+^ formation and upregulates cell proliferation (31, 32), while Blimp1 downregulates cell proliferation and instead promotes CD8^+^ effector memory cell function, indicating the Blimp1-BCL6 axis may also regulate CD8^+^ memory T cell responses (33, 34). Mice with a conditional deletion of Blimp1 in T cells were generated in the Calame lab by crossing the *Prdm1* ^F/F^ mice they originally made (20) to mice expressing either proximal Lck promoter^CRE^ or CD4 promoters-driven CRE expression transgenes, ultimately deleting Blimp1 in all T cell subsets (35, 36). Both *Prdm1* ^F/F^Lck^CRE^ and *Prdm1* ^F/F^CD4^CRE^ mice develop spontaneous colitis due to unrestrained inflammation caused by CD4^+^ T cells, establishing Blimp1 as a mitigator of exacerbated inflammatory responses (37). Similar results were obtained using a fetal liver RAG blastocyst complementation system in which mice lacking endogenous B and T lymphocytes (RAG1^-/-^ mice) were reconstituted with fetal liver cells from mice with a homozygous knock-in (KI) of a construct encoding a truncated Blimp1 protein that lacks the DNA binding domain (*Prdm1* ^GFP/GFP^ mice) (38). Further analysis of Blimp1-deficient CD4^+^ T cells *in vitro* showed that Blimp1 controls immune responses by repressing *Il2* transcription and consequently mitigating excessive T cell activation (36, 39). Of note, a recent study implicated Blimp1 as a potential regulator of pathogenic activity of tissue resident memory (T_RM_) CD4^+^ in murine models of intestinal inflammation (40), however, that study only compared wild type and double knockouts of both Blimp1 and Hobit, a Blimp1-related transcription factor previously shown to mediate the development of CD8^+^ T_RM_ cells in mice. The combined lack of Blimp1 and Hobit prevented the expression of pro-inflammatory cytokines, but the lack of single knockout mice for comparison makes it difficult to distinguish the contributions of Blimp1 and Hobit in this effect.

More recent studies focused on distinguishing Blimp1’s intrinsic roles in effector (T_EFF_) and regulatory (T_REG_) CD4^+^ T cells indicate that the severe phenotype of mice with T cell-specific deletion of Blimp1 is due to alterations in the function of both Foxp3^+^ T_REG_ and T_EFF_ cells, as mice with Foxp3^CRE^ -mediated deletion of Blimp1 does not fully recapitulate the phenotype of mice with T-cell specific deletion of Blimp1 (41, 42). Blimp1’s requirement for Foxp3^+^ T_REG_ cell function has been demonstrated in several different contexts and it is, at least in part, mediated by Blimp1’s non-redundant role in the induction of the regulatory cytokine IL-10.

In adipose tissue Foxp3^+^ T_REG_ cells, Blimp1 mitigates adipose tissue “beiging” and consequent protection from diet-induced obesity through the induction of IL-10 (43, 44). Blimp1 is also highly expressed in gut microbiota-associated RORγt^+^Foxp3^+^ T_REG_ cells, and in mice with a conditional deletion of Blimp1 in Foxp3^+^ T_REG_ cells, intestinal RORγt^+^Foxp3^+^ T_REG_ cells display a significant reduction in IL-10 expression and produce the inflammatory cytokine IL-17. This confers pathogenic properties to these cells, which have the ability to cause intestinal inflammation when adoptively transferred to RAG1^-/-^ mice (45). Further, ChIP assays demonstrated that Blimp1 directly binds to the *Il17* locus in Foxp3^+^ T_REG_ cells and directly represses *Il17* transcription (45). Central Nervous System (CNS) Foxp3^+^ T_REG_ cells also express Blimp1, and mice with a conditional deletion of Blimp1 in Foxp3^+^ T_REG_ cells displayed a significant reduction in IL-10 production and increased disease severity upon experimental autoimmunity encephalitis (EAE) induction. Blimp1’s actions in this model were described to depend on prevention of Foxp3 methylation (46).

In follicular regulatory T cells (T_FR_), which function to suppress germinal center immune responses and self-reactive antibody production (47, 48), Blimp1 maintains these immunosuppressive functions by directly repressing the *Il-23r* and *Cxcr5* genes and inducing *Il2ra*, *Ccr7*, and *Ctla-4* (49). Moreover, in Foxp3^-^ CD4^+^ T cells that produce both IL-10 and IFN-γ (T_R1_ cells), Blimp1 is required to maintain IL-10 production during parasitic infections, as shown in murine models (50). Although this Blimp1-dependent IL-10 production dampens an inflammatory response to the parasite, it mitigates tissue damage due to excessive inflammation. Overall, Blimp1 is most highly expressed in effector and regulatory T cell populations and crucially modulates the severity of CD4^+^ T cell-mediated inflammatory responses, notably by directly repressing the expression of cytokines, cytokine receptors and inducing IL-10 in several CD4^+^ T cell subsets.

In CD8^+^ T cells, Blimp1 has been shown to be strongly induced after LCMV infection and enables terminal effector differentiation (33). Mice with a conditional deletion of Blimp1 in CD8^+^ T cells, mediated by crossing *Prdm1* ^F/F^ mice with mice expressing CRE recombinase under human Granzyme B promoter control (GzB^CRE^), exhibit increased numbers of antigen-specific CD8^+^ effector cells after infection but stunted terminal effector differentiation, and Blimp1 was shown to mediate downregulation of IL-2 and control of effector responses and proliferation in CD8^+^ cells, further establishing the importance of Blimp1 in preventing unrestrained inflammatory responses to infection (33). Another study showed Blimp1 to be dispensable for the generation of CD8^+^ memory but essential for effective CD8^+^ T cell responses to viral infection, as both naïve and Influenza-primed Blimp1-deficient cells (using the fetal liver RAG blastocyst complementation system described above) exhibit stunted production of Granzyme B and effector differentiation in response to HKx31 viral infection (34). Further, Blimp1 was shown to repress Id3, an inhibitor of DNA-binding, and ultimately limit the formation of the CD8^+^ memory pool (51). Blimp1 has also been shown to be required for the generation of tissue-resident memory T cells (T_RM_) in several tissues, including the skin and the lung, and to promote Granzyme B production in CD8^+^ T_RM_ cells while limiting central memory populations (52–54).

### 2.3 Role of Blimp1 in Myeloid Cells

#### 2.3.1 The Role of Blimp1 in Controlling Antigen-Presentation and T Cell Priming by Dendritic Cells

Although the vast majority of the initial studies on Blimp1’s roles in immunity focused on its expression and function in lymphocytes, a role for Blimp1 in some myeloid cells has also been demonstrated (**Figure 3**). Studies have shown that conditional deletion of Blimp1 in dendritic cells (DC), in *Prdm1* ^F/F^ mice crossed with CD11c^CRE^ transgenic mice, led to heightened IL-6 production that was linked to autoantibody generation in female mice (55). In a different study, Blimp1 was shown to mitigate antigen-processing through direct repression of the *Ctss* gene, which encodes a cathepsin required for protein processing prior to presentation on MHC class II molecules (56). This was linked to expansion of the follicular helper T cell (T_fh_) repertoire and the ability to generate autoreactive antibodies in female mice (56). Further, transcriptomic analyses of human and murine intestinal DCs reported Blimp1 expression in a subset of CD103^+^ intestinal DCs, and mice with a conditional deletion of Blimp1 in DCs (mediated by CD11c^CRE^ mice crossed to *Prdm1* ^F/F^ mice) exhibit a significant reduction in this DC subset, indicating a role for Blimp1 in their differentiation (57). Additionally, this Blimp1^+^ DC subset in humans (CD103^+^SIRPα^+^ intestinal DCs) induced significantly more T_reg_ differentiation *in vitro* than other sorted intestinal DC populations, implicating these Blimp1^+^ DCs in the regulation of immune homeostasis in the intestines (57). These studies illustrate the importance of Blimp1 in regulating the differentiation and effector function of DCs, inhibiting exacerbated adaptive responses and ultimately preventing autoimmunity.

Blimp1 has also been implicated as a regulator of type I IFN production in DCs by a mechanism that involves control of IKKα and IRF7 activity by directly suppressing interleukin-1 receptor-associated kinase 3 (*Irak3*), a negative regulator of TLR signaling (58). In this study, it was shown that the same *Prdm1* ^F/F^ mice developed by the Calame group and crossed to CD11c^CRE^ transgenic mice display impaired responses to viral infection, consistent with decreased type I interferon production (58). These findings are seemly in contrast with earlier studies that demonstrated a role for Blimp1 in directly repressing IFN-β expression in sarcoma cells through the recruitment of repressive co-factors to the *IFN-β* locus (1, 7). However, the initial studies that implicated PRDI-BF1 (and another factor, PRDII-BF1) as direct repressors of type I IFN relied mostly on over expression and more recently, the same group showed that knockdown of PRDI-BF1 or PRDII-BF1 in mouse embryonic fibroblasts and human MG63 cells does not affect IFN-β repression after viral infection, indicating that at least in this system, Blimp1 is dispensable for the repression of IFN-β expression (59). Thus, it is conceivable that Blimp1 can only function as a repressor of *IFNB1* when overexpressed, which might or might not recapitulate Blimp1’s physiological role in repressing gene expression. As discussed below, it is conceivable that Blimp-1’s activity is, at least in part, regulated by its abundancy and the availability of different co-factors (7, 8, 60–63). In summary, Blimp1 can drive effector function in DCs and help to curtail excessive inflammation during an immune response through regulating T cell priming and activation.

#### 2.3.2 The Role of Blimp1 in Other Myeloid Cells

Although studies of Blimp1 function in the myeloid lineage are not nearly as extensive as those in lymphocytes, some of Blimp1’s targets and mechanisms of action have been determined to be conserved in both lineages. Similarly to that observed in T and B lymphocytes, Blimp1 and BCL6 act antagonistically to regulate differentiation and control homeostasis in osteoclasts, which are required for bone homeostasis (64). Moreover, in bone marrow-derived macrophages, Blimp1 has been shown to act as a transcriptional repressor of the chemokine CCL8, which was associated with regulation of the inflammatory response to *Listeria monocytogenes* infection in mice (65). Importantly, although Blimp1 expression was shown to be induced in bone-marrow derived macrophages by *Listeria monocytogenes* and Blimp1-deficient macrophages show higher *Ccl8* expression when compared to Blimp1-sufficient cells, comparative transcriptional analysis of Blimp1-sufficient or deficient bone marrow-derived macrophages by microarray analysis revealed a very low number of differentially expressed genes between the two groups, suggesting a limited role for Blimp1 in regulating gene expression in bone marrow-derived macrophages (65). Of note, LysM^CRE^- mediated gene deletion has been shown to be inconsistent before (66) and comparative analysis of the two different *Prdm1* probes in the microarray data deposited by the study above does indicate suboptimal deletion of *Prdm1* (see GSE53145). Nonetheless, this study also showed that *Prdm1* ^F/F^LysM^CRE+^ mice were less susceptible to *L. monocytogenes* infection and had increased CCL8 production along with heightened recruitment of γ/δ T cells to the peritoneal cavity (65).

Of note, despite the observation that peritoneal macrophages seemed to be affected by lack of Blimp1, the potential role of Blimp1 in other tissue-resident macrophages remains to be investigated. In recent years, tissue-resident macrophages have been shown to display distinct transcriptomic profiles depending on their tissue of residence and functional needs which are distinct from circulating monocytes, indicating a specific function for each tissue-resident macrophage population that differs from other monocytic cells (67). Thus, these cells might offer yet another interesting system to elucidate the intricate roles of Blimp1 in regulating gene expression and immune cell identity.

## 3 Regulation of Gene Expression by Blimp1

### 3.1 Blimp1 Is a Potent Repressor of Gene Expression

Comparative transcriptome analysis in Blimp1-sufficient and deficient immune cells illustrate the extensive role of Blimp1 in regulating gene expression. Blimp1 is thought to repress transcription by at least two different mechanisms: 1) competing with other transcriptional activators for direct binding to target loci and 2) directly binding and recruiting transcriptional co-repressors to the target locus to facilitate the formation of repressive chromatin structure (**Figure 2**). The first two of the five zinc fingers in the Blimp1 protein are sufficient for Blimp1 binding to target genes (6). The PR-domain of Blimp1 is crucial for the recruitment of chromatin-modifying co-factors, and this is illustrated by the inability of Blimp1β, a truncated isoform that lacks the intact PR-domain of full-length Blimp1α, to mediate repression of target gene expression, although it retains the zinc finger domains that facilitate DNA binding (4).

In addition to its target genes initially identified in B cells (discussed above), Blimp1 directly represses *Pax5*, *c-Myc*, and *Bcl6* to downregulate cell proliferation and promote terminal effector differentiation (21, 23, 24). Blimp1 also represses *c-Myc* in U937 and HL-60 human myeloid leukemia cells to downregulate cell proliferation (68). Similar to observations in plasma cell differentiation, Blimp1 also acts antagonistically with BCL6 in T lymphocytes to inhibit differentiation of follicular helper T (T_FH_) cells, and the antagonism between Blimp1 and BCL6 is also observed in the regulation of osteoclast differentiation in the bone marrow, indicating that Blimp1 can play conserved molecular functions in both lymphoid and myeloid lineages (**Table 1**) (30, 64).

**Table 1:** Conserved and unique targets of Blimp1 transcriptional regulation.

Gene	B Cells	T Cells	Natural Killer Cells	Myeloid	Repressor or Activator?
*ciita*	Yes	Not expressed	n.e.	n.e.	Repressor
*pax-5*	Yes	Not expressed	n.e.	Not expressed	Repressor
*igh*	Yes	Not expressed	Not expressed	Not expressed	Activator
*spiB*	Yes	n.e.	n.e.	n.e.	Repressor
*bcl6*	Yes	Yes	n.e.	Yes	Repressor
*c-myc*	Yes	No	n.e.	Yes	Repressor
*il6*	n.e.	n.e.	n.e.	Yes	Repressor
*Il10*	n.e.	Yes	n.e.	n.e.	Activator
*Il2*	n.e.	Yes	n.e.	n.e.	Repressor
*Tnf*	n.e.	n.e.	Yes	n.e.	Repressor

Reported direct targets of Blimp1 transcriptional regulation in immune cell subsets (*n.e., not evaluated).

In both CD4^+^ and CD8^+^ T cells, Blimp1 directly regulates the expression of several cytokine and chemokine genes. Of note, in a system in which both T_REG_ and T_EFF_ cells differentiate from the same pool of naïve CD4^+^ T cells *in vivo*, Blimp1 regulated both common and unique gene sets, illustrating its multifunctional role as a transcription factor (41). In naïve CD4^+^ T cells, antigen-specific TCR stimulation leads to expression of Blimp1 (35, 38), which directly represses the *Il2* and *Fos* genes, curtailing IL-2 expression and T cell proliferation (36). Similarly, during acute LCMV infection, Blimp1 is upregulated in effector and memory CD8^+^ T cells, and deletion of Blimp1 in these cells results in stunted terminal effector differentiation (33). In intestinal RORγt^+^ Foxp3^+^ regulatory T cells, Blimp1 directly binds and represses the *Il17* gene, preventing transcription and consequent inflammatory responses (45). This repression appears to involve Blimp1 competition with IRF4 for binding at the *Il17* locus, and mice with a T cell-specific deletion of Blimp1 show increased binding of IRF4 to this site and enhanced IL17 expression and Th17 differentiation (37, 45). Conversely, Blimp1 has also been shown to bind cooperatively with IRF4 in regulatory T cells to induce *Il10* expression, illustrating the nuanced nature of the molecular mechanisms of Blimp1 function even within the same immune cell populations and co-factors (69). In Foxp3^+^ T_reg_ cells, Blimp1 also protects and maintains Foxp3 expression by repressing transcription of Dnmt3a, a methyltransferase that represses Foxp3 transcription by methylating CNS2 of the *Foxp3* gene (46).

### 3.2 Blimp1 Can Compete With Transcriptional Activators and Recruit Chromatin-Modifying Protein Complexes

Although Blimp1 has not been shown to display any intrinsic chromatin modifying capabilities, despite having a histone methyltransferase-like domain characteristic of other members of the *Prdm1* family (24), one of the mechanisms by which it controls target gene transcription is through the recruitment of various chromatin modifying co-factors (**Figure 2**), including at least one lysine methyltransferase (see below). Blimp1 also recruits Groucho family proteins, which function as transcriptional co-repressors, including human Groucho-related gene (hGrg), which Blimp1 has been shown to recruit to facilitate repression of IFN-β in HeLa cells after Sendai virus infection (7).

The lysine methyltransferase G9a, also known as EHMT2, mediates the methylation of Histone 3 lysine 9 (H3K9) and Histone 3 Lysine 27 (H3K27) and consequent silencing of target gene transcription (70). Similarly, histone deacetylases (HDACs) confer repressive chromatin structure by deacetylating lysine residues at the N-terminal tails of target gene core histones (71, 72). ChIP assays have shown that Blimp1 directly binds the *IFN-β* gene and recruits G9a to this locus, consequently facilitating repressive chromatin modifications that silence *IFN-β* transcription (73). Blimp1 also directly binds HDAC1 and HDAC2, and HDAC recruitment by Blimp1 to the *c-Myc* promoter facilitates deacetylation of H3 at this site and consequent *c-Myc* repression in 293T human embryonic kidney fibroblasts and 18-81 murine pre-B cells (8, 21).

Protein arginine methyltransferase 5 (Prmt5) di-methylates arginine residues of target histones, and Blimp1 recruits Prmt5 in murine primordial germ cells to di-methylate H2A and H4, enabling proper germ cell development (61, 74). Lysine demethylase 1A (LSD1), also known as KDM1A, can demethylate both H3K4 and H3K9, and in this way LSD1 can act as both a co-repressor and co-activator. Blimp1 interacts with LSD1 through its proline-rich domain, which mediates downregulation of *Ciita*, *Pax5*, and *Spib*, further enforcing the terminally differentiated B cell transcriptional program (60). In CD8^+^ T cells, Blimp1 and LSD1 interaction is required to repress the expression of PD1 (*Pdcd1*) during the acute phase of viral infection (75). Similarly, enhancer of zeste 2 (EZH2) methylates H3K27 (H3K27me3), creating repressive chromatin structure of the target gene, and Blimp1 recruits EZH2 in plasma cells to downregulate *Spib*, *Tlr9*, *Klf2*, and *Btg1*, consistent with transcriptional signature of plasma cells (76). Non-POU domain containing octamer-binding protein (NONO) binds directly to the *Il6* gene, and NONO has been shown to act as a Blimp1 co-factor in monocyte-derived dendritic cells to suppress *Il6* transcription (77). Thus, in addition to directly competing with activators of transcription (45, 78), Blimp1 functions as a transcriptional repressor by recruiting chromatin modifying co-factors to target loci. Of note, Blimp1’s capability to recruit several different co-factors could be one of the mechanisms mediating its established multifaceted roles as both repressor and activator of transcription.

Additionally, Blimp1’s known DNA consensus binding motif has been show to resemble the consensus binding sites of some of the Interferon Regulatory Factors (IRF) family members, specifically IRF1 and IRF 2 (78) (**Figure 2**), which Blimp1 was shown to directly compete with for binding at the *IFN-β* locus in HeLa cells (78). Moreover, despite its potentially cooperative binding with IRF4 at the *Il10* locus in Foxp3^+^ T_REG_ cells, Blimp1 could potentially compete with IRF4 for binding at the *Il17a*/*f* CNS7 region in the same cells (45, 69). However, much remains to be learned about how Blimp1 cooperatively and competitively binds target loci, and as alluded to before, much of it may be enabled by the chromatin status at different loci, the availability of Blimp1 co-factors in different cellular contexts and the concentration of Blimp1 protein in different cell types, as at least during development, Blimp1 function was shown to be dose-dependent (63).

### 3.2 Blimp1 Can Also Directly Induce Gene Expression

Although Blimp1 was initially described as a transcriptional repressor, further studies uncovered its capability to function as a transcriptional activator (**Figure 2**). In addition to its repressive roles in B cells during plasmablast development, including direct repression of *Ciita*, *Pax5*, *Spib*, and *Id3*, Blimp1 also directly binds and activates several genes in pre-plasmablasts associated with enforcing the terminally differentiated plasmablast program, including genes involved in ER stress pathways, as activation of the UPR response is required for the ER expansion necessary for antibody production and secretion in plasmablasts (79). As shown by ChIP-seq, Blimp1 binds an enhancer region of immunoglobulin heavy chain (*Igh*) and activates transcription, continuing to promote the progression from pre-plasmablast to terminally differentiated plasmablast (79), however, the exact mechanism by which Blimp1 induce *Igh* expression in not clear.

In conjunction with IRF4, which has been shown to induce Blimp1 expression in lymphocytes (80, 81) Blimp1 directly promotes *Il10* transcription in Foxp3^+^T_REG_ cells, promoting an anti-inflammatory response (69). This was first observed in Foxp3^+^ T_reg_ cells in which Blimp1 was found to be required for *Il10* production, and Blimp1 and IRF4 both induce the Foxp3^+^T_REG_ effector transcriptional program, as shown by microarray gene expression analysis (69). Further ChIP studies revealed both Blimp1 and IRF4 bind to the *Il10* locus. In fact, as alluded to above, Blimp1 induces *Il10* expression in several T_REG_ cell subsets, including T_R1_ cells, adipose tissue T_REG_, CNS T_REG_, and RORγt^+^Foxp3^+^ intestinal T_REG_ cells (44–46, 50). Blimp1 also acts synergistically with c-Maf in Foxp3^-^ effector T cells to promote IL-10 production during *Toxoplasma gondii* infection, mitigating excessive inflammation and tissue damage (82). Thus, although Blimp1 was initially characterized as a transcriptional repressor, recent studies demonstrate that Blimp1 can act as both transcriptional repressor and activator, illustrating a multifaceted role for Blimp1 in gene regulation (**Figure 2**).

The mechanism (s) underlying Blimp1’s role as a transcriptional activator are far less understood than the ones regulating Blimp’s repression function. Blimp1’s synergistic partnership with IRF4 and c-Maf has not been studied in detail, e.g., it remains to be determined if and how Blimp1 physically interacts with these and other factors or if both molecules just bind to DNA in close proximity to each other. Given the ample repertoire of transcriptional regulators that Blimp1 can partner with, including chromatin modifiers such as LSD1, which can mediate both repressing and activating chromatin modifications, it is likely that differential physical interaction/recruitment of co-factors mediates Blimp1 apparent opposite function in regulating gene expression. However, this remains to be examined in detail.

## 4 Blimp1 and Regulation of Human Disease

Consistent with the established role of Blimp1 as an essential regulator of immune cell function (**Figure 3**), numerous disease-linked single nucleotide polymorphisms (SNP) have been associated with the *PRDM1* gene. *PRDM1-*associated risk alleles have been linked with ulcerative colitis and Crohn’s disease through a comprehensive meta-analysis of six genome-wide association studies (GWAS) on ulcerative colitis patients, and another study identified one particular SNP, Ser354Asn, located at position 106659789 on chromosome 6, is associated with decreased plasmablasts, increased T cell proliferation and increased IFN-γ production (83, 84). *PRDM1* risk alleles have also been associated with systemic lupus erythematosus (SLE), and females carrying the rs548234 *PRDM1* SNP, located at position 106120159 on chromosome 6, display a reduction in *PRDM1* expression in monocyte-derived dendritic cells (Mo-DCs) but not B cells in the peripheral blood (85). Meta-analysis of rheumatoid arthritis GWAS studies also revealed the presence of the rs548234 SNP as a risk allele, further suggesting the implication of *PRDM1* in autoimmune disease pathogeneis (86).

Additionally, *PRDM1* has been identified as a tumor suppressor gene and found to be inactivated in several lymphomas, including natural killer cell lymphoma (NKCL), diffuse large B cell lymphoma (DLBCL), and anaplastic large T-cell lymphoma (ALCL) (87–89). Further, BCL6 expression has been shown to be unrestrained in some lymphomas, and it has been proposed that this mediates downregulation of *PRDM1* and consequent cancer progression due to a dysregulated Blimp1-BCL6 axis (26, 87). Taken together, these studies suggest potentially crucial roles for Blimp1 in both lymphoid and myeloid lineages in humans. In human natural killer (NK) cells, *PRDM1* is induced after stimulation both *in vitro* and *in vivo*, and *PRDM1* directly binds and suppresses *IFNG* and *TNF* transcription, potentially restraining a prolonged inflammatory response by NK cells (90). However, mechanisms of *PRDM1* function in other human immune cells are not fully explored and may be crucial to understanding autoimmune disease pathogenesis and cancer. Importantly, studies focusing on the potential functional consequences of *PRDM1*-associated disease-linked SNP have so far only established association between these variants and altered immune cell function, and no direct causal roles have been attributed to *PRDM1*-associated variants in human disease.

## 5 Discussion and Future Directions

Although the functions of Blimp1 have been extensively studied in B and T lymphocytes, the underlying molecular mechanisms of action have not been fully elucidated and require further studies. Overall, the mechanisms underlying regulation of gene expression by Blimp1 are complex and likely nuanced, including the dependence on Blimp1 protein concentrations and the reliance on the availability of various co-factors and other transcriptional regulators. This supports the notion that Blimp1 function is context-dependent and might vary depending on the particular immune cell subset and tissue microenvironment. However, the potential role of Blimp1 in regulating tissue homeostasis and immunity is only now beginning to be explored. Studies focusing on this aspect of Blimp1’s biology have so far focused solely on T cells, specifically T_RM_ (40, 52–54). In the myeloid compartment, Blimp1 studies in specific immune cell subsets *in vivo* are still very few and mostly limited to bone marrow-derived myeloid cells, only rarely focused on splenic and blood-monocyte-derived DC (55, 56) or peritoneal macrophages (65). The recent understanding of the vast heterogeneity of tissue-resident myeloid populations by high-throughput sequencing studies highlighting the distinct tissue-imprinted transcriptomic and epigenomic profiles of tissue-resident myeloid cells (67) supports the notion that the role of transcription factors in myeloid cell homeostasis and function *in vivo* goes far beyond what can be learned from studies of *in vitro*-derived myeloid cells (91, 92). Considering this, it is likely that the role of Blimp1 in regulating myeloid cell function and potentially overall tissue immune homeostasis is far more extensive than what has been described so far, and moving forward, the field would greatly benefit from further studies on Blimp1’s function in tissue-resident immune cells, which has the potential to further our understanding of Blimp1’s biology in different systems and its implications in human disease.

## Author Contributions

SN and GM wrote this manuscript. SN made all the figures. All authors contributed to the article and approved the submitted version.

## Funding

This work was supported by NIH-NIAID (5R01AI103542, 1R01AI127406 and R21AI151987 to GM), by the CSMC Dept. of Biomedical Sciences “Leon Fine Translational Award” (to GM) and by NIH (T32-GM118288 to SN).

## Conflict of Interest

The authors declare that the research was conducted in the absence of any commercial or financial relationships that could be construed as a potential conflict of interest.

## Publisher’s Note

All claims expressed in this article are solely those of the authors and do not necessarily represent those of their affiliated organizations, or those of the publisher, the editors and the reviewers. Any product that may be evaluated in this article, or claim that may be made by its manufacturer, is not guaranteed or endorsed by the publisher.
